# Asymmetric Thermally Activated Delayed Fluorescence Materials With Aggregation-Induced Emission for High-Efficiency Organic Light-Emitting Diodes

**DOI:** 10.3389/fchem.2020.00049

**Published:** 2020-02-26

**Authors:** Huanhuan Li, Yibin Zhi, Yizhong Dai, Yunbo Jiang, Qingqing Yang, Mingguang Li, Ping Li, Ye Tao, Hui Li, Wei Huang, Runfeng Chen

**Affiliations:** ^1^Key Laboratory for Organic Electronics and Information Displays & Jiangsu Key Laboratory for Biosensors, Jiangsu National Synergetic Innovation Center for Advanced Materials (SICAM), Institute of Advanced Materials (IAM), Nanjing University of Posts and Telecommunications, Nanjing, China; ^2^Institute of Flexible Electronics, Northwestern Polytechnical University, Xi'an, China

**Keywords:** thermally activated delayed fluorescence, asymmetric structure, aggregation-induced emission, charge-transfer, electroluminescence

## Abstract

The exploitation of thermally activated delayed fluorescence (TADF) emitters with aggregation-induced emission is highly prerequisite for the construction of highly efficient electroluminescent devices in materials science. Herein, two asymmetric TADF emitters of **SFCOCz** and **SFCODPAC** with charming aggregation-induced emission are expediently designed and prepared based on highly twisted strong electron-withdrawing acceptor (A) of sulfurafluorene (**SF**)-modified ketone (**CO**) and arylamine donor (D) in D_1_−A–D_2_ architecture by simple synthetic procedure in high yields. High photoluminescence quantum yields up to 73% and small singlet–triplet splitting of 0.03 eV; short exciton lifetimes are obtained in the resultant molecules. Strikingly, efficient non-doped and doped TADF organic light-emitting diodes (OLEDs) facilitated by these emitters show high luminance of 5,598 and 11,595 cd m^−2^, current efficiencies (CEs) of 16.8 and 35.6 cd/A, power efficiencies (PEs) of 9.1 and 29.8 lm/W, and external quantum efficiencies (EQEs) of 7.5 and 15.9%, respectively. This work furnishes a concrete instance in exploring efficient TADF emitter, which is highly conducive and encouraging in stimulating the development of TADF OLEDs with high brightness and excellent efficiencies simultaneously.

## Introduction

Luminescent materials that are capable of thermally activated delayed fluorescent (TADF) have been widely investigated not only because of their great potential in utilizing theoretically 100% internal quantum efficiency (IQE) through the back transfer of non-radiative triplet exactions (75%) into radiative singlet excitons conferred by small single–triplet energy splitting (Δ*E*_ST_) for efficient reverse intersystem crossing (RISC), but also due to their fundamental significance both in scientific investigations and technological applications of organic electronics (Uoyama et al., 2012; Tao et al., 2014; Etherington et al., 2016; Guo et al., 2018; Han et al., 2018; Kotadiya et al., 2019; Pershin et al., 2019; Zhang Y. L. et al., 2019). With flourish developments over the past few years, considerable attention has been devoted to designing and exploiting excellent TADF materials with the consideration of the following rational metrics (Park et al., 2016; Chen et al., 2017; Im et al., 2017; Wong and Zysman-Colman, 2017; Yang Z. et al., 2017; Zhang Y. et al., 2019): (i) the separated highest occupied molecular orbital (HOMO) and lowest unoccupied molecular orbital (LUMO) distributions for enabling small Δ*E*_ST_; (ii) slight overlap of frontier molecular orbital (FMO) for maintaining high photoluminescence quantum yield (PLQY); (iii) short exciton lifetimes to eliminate the concentration-induced quenching effect; (iv) reduced intermolecular interactions to alleviate aggregation quenching processes; and (v) acceptable thermal stability for long-term device operation and ease synthesis procedure for mass productions. Nonetheless, most of the reported TADF materials suffer from serious aggregation caused quenching (ACQ) phenomenon that obviously hinders their practical applications (Einzinger et al., 2017; Wei et al., 2017, 2019; Wong and Zysman-Colman, 2017; Gan et al., 2019). Therefore, it remains forbidden issues to construct prominent TADF materials because of the great challenge in obtaining such aforementioned features in a molecule simultaneously, especially for alleviating the serious ACQ (Aydemir et al., 2017).

Aggregation-induced emission (AIE) is a fascinating optical phenomenon with greatly enhanced luminescent efficiency in solid state, which has been increasingly emerging as a promising candidate in organic electronics, bio-electronics, and photonics (Huang et al., 2017; Mao et al., 2017; Tsujimoto et al., 2017; Yang J. et al., 2017; Chen et al., 2019). The implantation of AIE properties into TADF materials has demonstrated a possibility to suppress the ACQ of solid films (Lee et al., 2017; Zheng et al., 2019). Generally, the linkage of donor and acceptor units through the spiro- and/or twist structure to lessen the overlap of FMO and to inhibit the molecular aggregation has been proven to be a perspective strategy for achieving AIE-TADF materials. Following this guideline, an extensive collection of AIE-TADF materials has been designed and explored in fabricating efficient TADF organic light-emitting diodes (OLEDs). Recently, diphenyl ketone, which can not only serve as an electron-deficient core to construct charge transfer (CT) molecule with spatially separated HOMO and LUMO distributions through the incorporation of varieties of donor units for achieving a small Δ*E*_ST_ but can also be used as the twist and rotation center to reduce the interactions for alleviating the self-quenching effect of multiple molecules and to incorporate AIE for boosting emission of the resultant materials in solid state, has been regarded as one of the most key building blocks in constructing TADF materials with the AIE character (Guo et al., 2017; Huang et al., 2017). These impressive advantages have stimulated us to explore new diphenyl ketone-based TADF derivatives for fabricating high-performance OLEDs.

**Figure 1:**
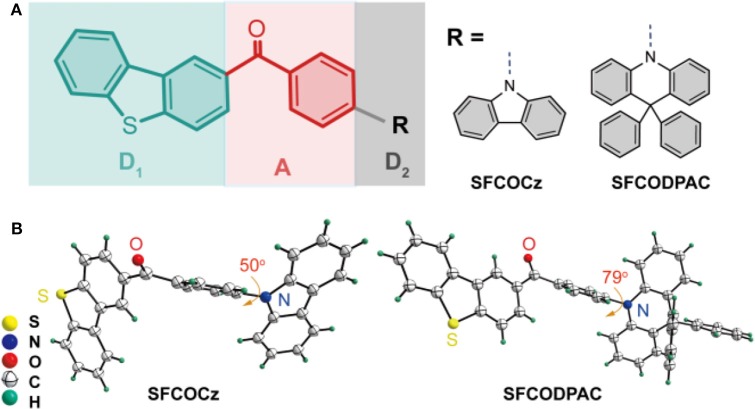
**(A)** Schematical drawing of designed **SFCO**-based thermally activated delayed fluorescence (TADF) materials with asymmetric D_1_−A–D_2_ architecture. **(B)** ORTEP single-crystal structures of **SFCOCz** (CCDC 1964730) and **SFCODPAC** (CCDC 1964747).

Herein, to achieve the AIE-TADF materials, we designed and synthesized two emitters of **SFCOCz** and **SFCODPAC** with asymmetric D_1_−A–D_2_ architecture (Figure 1A) through the direct linkage of sulfurafluorene (**SF**)-modified ketone (**CO**) and arylamine of carbazole (**Cz**) or 9,9-diphenyl-9,10-dihydroacridine (**DPAC**). In this moiety, the D_1_−A–D_2_ can effectively render separated HOMO and LUMO distributions to guarantee a small Δ*E*_ST_ for the promotion of RISC process under thermal activation; moreover, the twisted and asymmetric molecular configuration can endow AIE and restrain intermolecular interaction of π-π stacking and/or aggregation to reduce ACQ in solid state (Aydemir et al., 2017; Wang et al., 2017; Zeng et al., 2018; Liu et al., 2019). These newly constructed AIE-TADF materials in D_1_−A–D_2_ skeleton can be easily prepared with high yields up to 68%. Strikingly, **SFCOCz** and **SFCODPAC** exhibited excellent TADF with small Δ*E*_ST_ of ~0.03 eV, high PLQY (Φ_PL_) of ~73%, and efficient RISC rate and relatively short delayed fluorescence lifetime. High-performance non-doped and doped TADF OLEDs endowed by these emitters were fabricated, showing high luminance of 5,598 and 11,595 cd m^−2^ and external quantum efficiencies (EQEs) of 7.5 and 15.9%, respectively. The ease of synthetic route, excellent optoelectronics, and high device performance make the **SFCO**-based asymmetric TADF emitters promising candidates in practical applications, conferring a new paradigm for next-generation organoelectronics.

## Results and Discussion

### Design, Synthesis, and Characterization

To prepare the asymmetric TADF materials exhibiting AIE trait, two molecules, namely (4-(9*H*-carbazol-9-yl)phenyl)(dibenzo[b,d]thiophen-2-yl)methanone (**SFCOCz**) and dibenzo[b,d]thiophen-2-yl(4-(9,9-diphenylacridin-10(9*H*)-yl)phenyl)methanone (**SFCODPAC**), were designed and synthesized in D_1_−A–D_2_ architecture through the direct connection of sulfurafluorene-tailored diphenyl ketone and donor of Cz (**SFCOCz**) or DPAC (**SFCODPAC**) by a conventional two-step procedure composed of Friedel–Crafts acylation and C–N coupling reaction (Figure 1A and Scheme S1, Figures S1–S4). As revealed by single-crystal X-ray diffraction (XRD) analysis, the dihedral angle between arylamine and **SFCO** in these two molecules is up to 79° (Figure 1B and Table S1). Such a highly twisted molecular conformation would not only be beneficial to reduce electron communications, ensuring an optimized HOMO and LUMO separation for acquiring a small Δ*E*_ST_, but can also effectively suppress the molecular aggregations in solid state for eliminating ACQ (Wang et al., 2017). **SFCOCz** and **SFCODPAC** display high thermal stabilities (Figure S5), exposing the decomposition temperatures (*T*_d_) of 386 and 429°C as revealed by the thermogravimetric (TGA) measurements and melting temperature (*T*_m_) of 197 and 251°C as measured by differential scanning calorimetry (DSC) analyses. The slightly higher *T*_d_ and *T*_m_ of **SFCODPAC** than those of **SFCOCz** could be well-explained by its high molecular weight and rigid structure. In addition, the vacuum-evaporated thin films on glass substrates are amorphous and uniform with quite small root-mean-square roughness (RMS) of 0.307 and 0.173 nm for (Figure S6) **SFCOCz** and **SFCODPAC**. The excellent thermal and morphology stabilities of resultant AIE-TADF materials would be favored for vacuum-deposited device fabrication and long-term operation stability.

### Photophysical Properties

**Figure 2:**
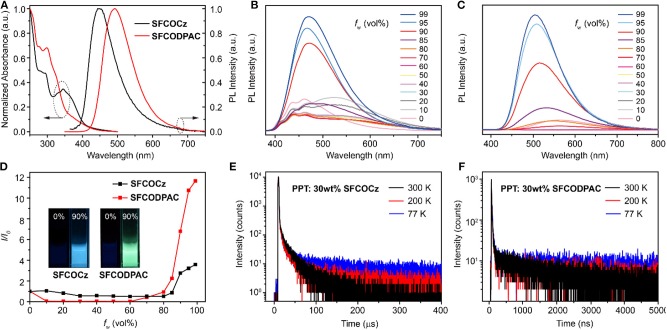
**(A)** Absorption and photoluminescence (PL) (excited at 320 nm) spectra of asymmetric thermally activated delayed fluorescence (TADF) molecules in films. PL spectra of **(B) SFCOCz** and **(C) SFCODPAC** in tetrahydrofuran (THF)/water mixtures with varied water fractions (*f*_w_). **(D)** Plots of *I*/*I*_0_ values vs. water fractions (*f*_w_). *I*_0_ is representing the integrated intensity in THF. Inset: photographs of the TADF molecules in THF/water mixture. **(E,F)** Temperature-dependent decay profiles of **(E) SFCOCz** and **(F) SFCODPAC** in doped film (30 wt% in PPT).

The photophysical profiles of asymmetric TADF molecules **SFCOCz** and **SFCODPAC** in dilute dichloromethane solution (CH_2_Cl_2_, 1 × 10^−5^ mol L^−1^) and neat and doped films (Figure 2 and Figures S7–S11) were detailedly investigated by UV–visible absorption and photoluminescence (PL) spectra. **SFCOCz** and **SFCODPAC** imply that the *n*–π^*^ transition dominated absorption band peaked ~300 nm, and the CT band at ~350 nm originated from intramolecular CT (ICT) from arylamine to **CO** (Table 1 and Table S2) (Lee et al., 2017). The PL spectra of **SFCOCz** and **SFCODPAC** exhibit typical structureless ICT fluorescence bands located at 484 and 558 nm in CH_2_Cl_2_, respectively. The ICT characters were also demonstrated by the red-shifted emission bands with the increasing in solvent polarities (Figure S8). In film states, these two molecules reveal nearly the same absorption profiles to those of the solution, showing absorption peaks at 295 and 346 nm for **SFCOCz** and 297 and 359 nm for **SFCODPAC**. These findings verify the effective suppressing of molecule aggregation in the films. The optical bandgaps (^°pt^*E*_g_s), calculated by the onset edge of the absorption spectra, were 3.04 and 2.88 eV for **SFCOCz** and **SFCODPAC**, respectively. For the PL spectra, **SFCOCz** and **SFCODPAC** also show structureless ICT bands at ~450 and 492 nm with corresponding Φ_PL_ of ~22 and 73%, respectively. Notably, the Φ_PL_s of **SFCOCz** and **SFCODPAC** in the solid state are up to ~3.7-fold that in the solution, indicating the obvious AIE characteristics. To further demonstrate the AIE properties of these two TADF molecules, the PL spectra in tetrahydrofuran (THF) with varied water fractions (*f*_w_) were performed. **SFCOCz** and **SFCODPAC** exhibit intense emissions as the formation of nanoaggregates upon injecting a large amount of poor solvent of H_2_O (fraction >80%) into THF solutions (Figures 2B–D), suggesting again the AIE features. This significantly enhanced photoluminescence in nanoaggregates should be due to the suppressed molecule rotation and motion that are highly active in the solution, thus blocking the non-radiative decay of excitons. In addition, we also explore the PL properties of **SFCOCz** (30 wt%) and **SFCODPAC** (30 wt%) doped films using 2,8-bis(diphenyl-phosphoryl)-dibenzo[b,d]thiophene (PPT) as the host material (Figure S9). Because of the strong polarity of PPT, the doped films showcase the red-shifted emission peaks with lower Φ_PL_ compared to their corresponding neat films (Méhes et al., 2014).

**Table 1:** Optical, thermal, and electrical properties of **SFCOCz** and **SFCODPAC**.

**Compound**	***T*_**m**_/*T*_**d**_ (^**°**^C)**	*****λ*****_****abs****_ **(nm)**		**opt *E*_g_ (eV)**	*****λ*****_****em****_ **(nm)**		**CV (eV)**
		**CH_**2**_Cl_**2**_**	**Film**		**CH_**2**_Cl_**2**_**	**Film**	**HOMO/LUMO**
SFCOCz	197/386	273, 293, 328, 342	283, 295, 331, 346	3.04	484	449	−5.70/−2.66
SFCODPAC	251/429	273, 295, 315, 360	276, 297, 322, 359	2.88	558	492	−5.52/−2.64

To demonstrate their TADF properties, a set of experiments was carried out. We attempted to estimate the Δ*E*_ST_ of **SFCOCz** and **SFCODPAC** on the basis of the fluorescence spectra and phosphorescence spectra at 77 K. As shown in Figure S10, the Δ*E*_ST_s of **SFCOCz** and **SFCODPAC** in neat and (30 wt%) doped films were 0.17 and 0.03 eV in the neat films, and 0.21 and 0.02 eV in the doped films, respectively, potentially enabling the process of RISC by thermal activation. The transient photoluminescence profiles were also performed to understand the photophysical process of **SFCOCz** and **SFCODPAC** (Figures 2D,E and Figure S11). The double exponential lifetime decay curve was observed in both of **SFCOCz** and **SFCODPAC** in neat and doped films, showing a short nanosecond lifetime of prompt fluorescence (τ_PF_) and a microsecond lifetime (τ_DF_) of delayed emission (Table S2). Contributed by the small Δ*E*_ST_, τ_DF_ of **SFCODPAC** were 0.22 and 0.84 μs in the neat and doped films, respectively, which is 1,513- and 24-fold smaller than those of **SFCOCz**. The temperature-dependent lifetime measurements of the doped films further present efficient proof of the TADF characteristics of these two materials. The delayed component afforded by the RISC was increased gradually, with the temperature increasing from 77 to 300 K (Figures 2E,F), obviously indicating the TADF trait. By means of the PLQY and lifetime, the calculated rate of RISC (Supplementary Material) are 0.1 × 10^5^ and 6.16 × 10^6^ s^−1^ in neat films, and 1.3 × 10^5^ and 2.16 × 10^6^ s^−1^ in doped films for **SFCOCz** and **SFCODPAC**, respectively. The greatly improved RISC of **SFCODPAC** could be attributed to its smaller Δ*E*_ST_.

### Theoretical and Electrochemical Investigations

**Figure 3:**
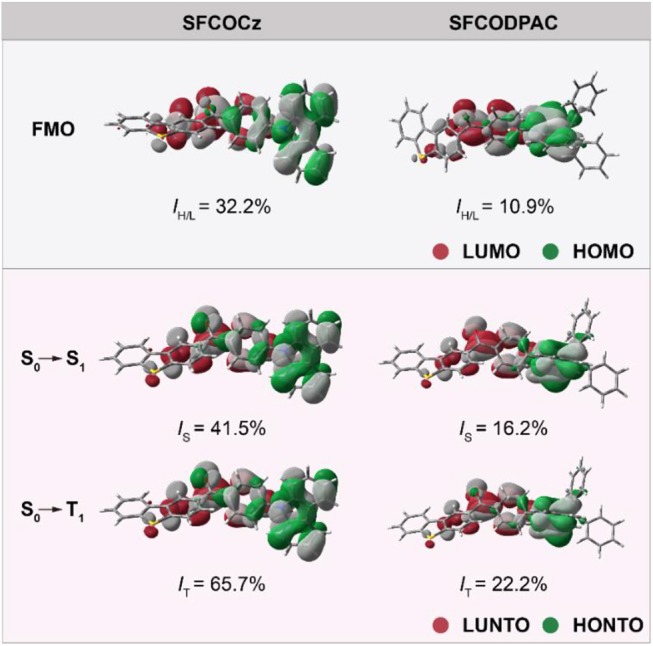
Density functional theory (DFT) calculated frontier molecular orbital (FMO) distributions, natural transition orbital (NTO) analyses, and *I*_H/L_, *I*_S_, and *I*_T_ of asymmetric thermally activated delayed fluorescence (TADF) molecules of **SFCOCz** and **SFCODPAC**.

As shown in Figure 3, the spatial distributions of HOMOs and LUMOs of **SFCOCz** and **SFCODPAC** were clearly observed (Leitl et al., 2014). The HOMOs are largely located on the electron-donating (D_2_) unit of **Cz** for **SFCOCz** and **DPAC** for **SFCODPAC**, while the LUMOs are mainly concentrated on **CO** and slightly located on the **SF** unit, showing an overlap extent (*I*_H/L_) of 32.2 and 10.9%, respectively (Chen et al., 2015). The discrete HOMOs and LUMOs distribution in the asymmetric D_1_−A–D_2_ materials would be favorable for realizing small Δ*E*_ST_. The simulated Δ*E*_ST_s were 0.38 and 0.01 eV for **SFCOCz** and **SFCODPAC** in the monomeric state. The decreased Δ*E*_ST_ of **SFCODPAC** may be originated from the combined effect of the strong electron-donating ability of **DPAC** and large dihedral angle between **CO** and **DPAC** for more separated FMO distributions. The FMO energy levels of these two molecules were analyzed by the cyclic voltammetric (CV) curves. According to the oxidation onset at 0.94 and 0.76 V, the HOMOs were evaluated to be −5.70 and −5.52 eV for **SFCOCz** and **SFCODPAC**, respectively. By means of optical bandgaps and HOMOs, the LUMOs were speculated to be −2.66 and −2.64 eV. To reveal the electron transition components of excited states in **SFCOCz** and **SFCODPAC**, the natural transition orbital (NTO) analyses were also carried out (Li et al., 2018). The highest occupied NTO (HONTO) and the lowest unoccupied NTO (LUNTO) distributions of **SFCOCz** and **SFCODPAC** at excited states are almost identical to their corresponding ground-state FMOs. The HONTOs of these two molecules were primarily dominated by the donor moiety; the LUNTO were largely assigned on the **CO** core with slight extension on the **SF** unit. The overlap extent of HONTO and LUNTO is 41.5 and 16.2% on singlet state (*I*_s_), and 65.7 and 22.2% on triplet state (*I*_T_) for **SFCOCz** and **SFCODPAC**, respectively. The overlap at the excited state should play an important role in guaranteeing high PLQY.

### Electroluminescent Performance of OLEDs

**Figure 4:**
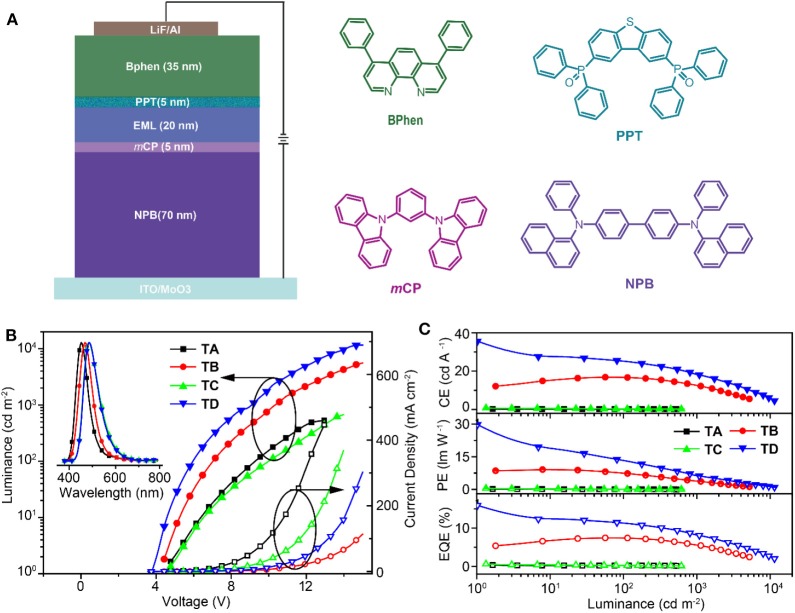
**(A)** Device configuration and chemical structures of the adopted materials, **(B)** luminance (solid symbols)–current density (open symbols)–voltage inset: electroluminescence (EL) spectra of thermally activated delayed fluorescence organic light-emitting diodes (TADF OLEDs) at 6.0 V, and **(C)** efficiencies–luminescence curves of the non-doped and doped TADF OLEDs.

To further elucidate the feasibility of asymmetric D_1_−A–D_2_ molecules in constructing high-performance thermal-evaporated devices, the non-doped (TA-TB) and doped (TC-TD) TADF OLED of **SFCOCz** (TA, TC) and **SFCODPAC** (TB, TD) were successfully fabricated using the following configurations (Figure 4A): ITO/MoO_3_ (30 nm)/4,4′-bis[*N*-(1-naphthyl)-*N*-phenylamino]-1,1′-biphenyl (NPB) (50 nm)/1,3-bis(carbazol-9-yl)benzene (*m*CP) (5 nm)/EML (20 nm)/PPT (5 nm)/bathophenanthroline (BPhen) (5 nm)/LiF (1 nm)/Al (100 nm). In these devices, NPB and BPhen were hole- and electron-transporting layers, and *m*CP and PPT were exciton-blocking layers. TA-TD exhibited pure electroluminescence (EL) spectra inherited from their neat and doped films at different driving voltages, showing maximum emission peaks at 451, 482, 468, and 485 nm with corresponding Commission International de l'Eclairage (CIE) coordinates of (0.16, 0.11), (0.20, 0.37), (0.15, 0.18), and (0.19, 0.37), respectively (Table S3). Compared to **SFCOCz**-based TA and TC, **SFCODPAC**-endowed TADF OLEDs of TB and TD revealed slightly decreased driving voltages (*V*_on_s) of 4.4 and 3.8 V, respectively. In addition to the decreased *V*_on_, TB and TD also demonstrated improved luminance up to 5,598 and 11,595 cd m^−2^ than those of TA and TC. The low *V*_on_s and high luminance of TB and TD should be attributed to its high HOMO and PLQYs of **SFCODPAC** for the promotion of hole injection from the adjacent layer and the generation of excitons. Not surprisingly, TADF OLEDs based on **SFCODPAC** show much better device efficiencies with current efficiencies (CEs) of 16.8 and 35.6 cd/A, power efficiencies (PEs) of 9.1 and 29.8 lm/W, and EQEs of 7.5 and 15.9% for TB and TD, respectively (Figures 4B,C and Table S3). There values are comparable to the best results of non-doped and doped TADF OLEDs based on AIE-type TADF emitters (Table S4). In addition, TD displays acceptable device performance with 29.1 and 21.0 cd A^−1^ for CE, 11.8 and 5.6 lm W^−1^ for PE, and 13.1 and 9.4% for EQE at 100 and 1,000 cd m^−2^, respectively. Although the non-doped films suggest enhanced PLQYs, the poor charge transport properties in neat films induced by highly twisted asymmetric molecular skeleton should be responsible for the decreased device performance of non-doped TADF OLEDs.

## Conclusions

In summary, we have succeeded in designing and developing two asymmetric TADF molecules of **SFCOCz** and **SFCODPAC** in the D_1_−A–D_2_ skeleton through a simple and effective procedure in high yields. The resultant TADF molecules exhibit small Δ*E*_ST_, short delayed lifetimes, and robust AIE characteristics with high PLQY up to 73%. More impressively, benefiting from the excellent optoelectronic properties, the non-doped and doped TADF OLEDs conferred by **SFCODPAC** display high efficiencies with peak EQEs of 7.5 and 15.9%, accompanied with the CIE coordinates of (0.20, 0.37) and (0.19, 0.37), respectively. Our work here provides a delicate molecular design strategy for the construction of asymmetric AIE-type TADF emitters, and clearly manifests the significant advance of the combined TADF and AIE features in exploiting high-performance organic emitters.

## Data Availability Statement

The datasets generated for this study can be found in the Cambridge Structural Database under the following identifiers: CCDC/1964730, CCDC/1964747.

## Author Contributions

HuaL, YZ, YT, WH, and RC conceived the experiment. HuaL, YZ, YJ, and HuiL performed the characterizations of optoelectronic properties. YD, YT, and ML designed and fabricated the devices. QY and PL contributed the theoretical simulations. HuaL, YZ, YJ, and YT wrote the manuscript. All authors contributed to the scientific discussion.

### Conflict of Interest

The authors declare that the research was conducted in the absence of any commercial or financial relationships that could be construed as a potential conflict of interest.
