# Supplementary material for: Asymmetric Thermally Activated Delayed Fluorescence Materials With Aggregation-Induced Emission for High-Efficiency Organic Light-Emitting Diodes
Source: Front Chem. 2020 Feb 26;8:49. doi: 10.3389/fchem.2020.00049 (PMC7054483; doi:10.3389/fchem.2020.00049)
Supplement: Supplementary file 1 [file Data_Sheet_1.docx]

Supplementary Material

**Asymmetric Thermally Activated Delayed Fluorescence Materials with Aggregation-Induced Emission for High-Efficiency** **Organic Light-Emitting Diodes**

*Huanhuan Li^1^, Yibin Zhi^1^, Yizhong Dai^1^, Yunbo Jiang^1^, Qingqing Yang^1^, Mingguang Li^1^, Ping Li^1^, Ye Tao^1^*, Hui Li^1^, Wei Huang^1, 2^, Runfeng Chen^1*^*

^1^Key Laboratory for Organic Electronics and Information Displays & Jiangsu Key Laboratory for Biosensors, Institute of Advanced Materials (IAM), Jiangsu National Synergetic Innovation Center for Advanced Materials (SICAM), Nanjing University of Posts & Telecommunications, 9 Wenyuan Road, Nanjing 210023, China

^2^Institute of Flexible Electronics, Northwestern Polytechnical University, Xi’an, 710129, China.

**CONTENT**

[1 Instrumentation and Materials 2](#_Toc29668810)

[2 Single Crystal Analysis 6](#_Toc29668811)

[3 Thermal Properties 7](#_Toc29668812)

[4 Morphology Properties 8](#_Toc29668813)

[5 Photophysical Properties 9](#_Toc29668814)

[6 Calculation of the Photophysical Rate Constants. 12](#_Toc29668815)

[7 Theoretical Calculations 14](#_Toc29668816)

[8 Electrochemical Properties 15](#_Toc29668817)

[9 Device Fabrications and Measurements 16](#_Toc29668818)

[10 References 19](#_Toc29668819)

# Instrumentation and Materials

**Materials:** All reagents, unless otherwise specified, were purchased from Aldrich, Acros or Alfa Aesar, and used without further purification. Manipulations involving air-sensitive reagents were performed in an atmosphere of dry argon.

**Characterization methods:** ^1^H and ^13^C-nuclear magnetic resonance (NMR) spectra were recorded on a Bruker Ultra Shield Plus 400 MHz instrument with CDCl_3_ as the solvent and tetramethylsilane (TMS) as the internal standard. The quoted chemical shifts are in *ppm* and the *J* values are expressed in Hz. The splitting patterns have been designed as follows: s (singlet), d (doublet), t (triplet), dd (doublet of doublets) and m (multiplet). The molecular weight was measured using a Bruker matrix-assisted laser desorption/ionization time of flight mass spectrometer (MALDI-TOF MS) with trans-2-[3-(4-tert-butylphenyl)-2-methyl-2-propenylidene]malononitrile (DCTB) as the matrix. The elementary analysis was performed on vario ELcube.


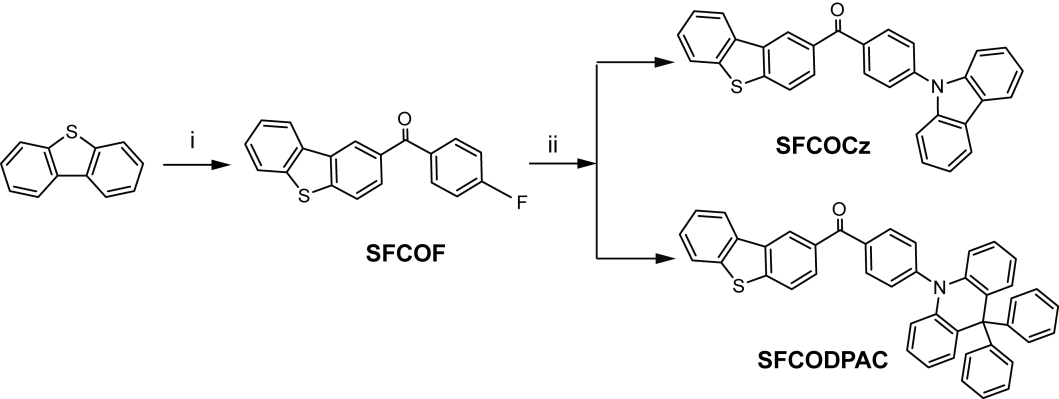


**Scheme S1.** The synthetic route of the molecules: (i) 4-fluorobenzoyl chloride, AlCl_3_, DCM, 0ºC to rt, 3 h; (ii) 1.2 equivalent: *9H*-carbazole (**SFCOCz**) or 9, 9-diphenyl-9, 10-dihydroacridine (**SFCODPAC**), *t*-BuOK, DMF, 110ºC, overnight.

**Synthesis of dibenzo[b, d]thiophen-2-yl(4-fluorophenyl)methanone (SFCOF)**

Dibenzo[b, d]thiophene (1.8 g, 10.0 mmol), anhydrous aluminum chloride (8.0 g, 60.0 mmol) were dissolved in anhydrous dichloromethane (DCM) (50 mL) under argon protection and then the mixture was cooled to 0^o^C by an ice/water bath. 4-fluorobenzoyl chloride (1.5 mL, 12.0 mmol) was added into the mixture slowly to give a bright yellow solution. The reaction solution was allowed to be stirred at 0°C for 15 min, and followed by stirring at room temperature for 3.0 h. Then, the reaction was quenched with water (25 mL) and extracted with dichloromethane (CH_2_Cl_2_) for three times (3×200 mL). The organic layer was collected and dried with anhydrous sodium sulfate (Na_2_SO_4_). After the evaporation of the solvent, the solid residue was purified by column chromatography. Yield: 2.47 g of white powder (81%). ^1^H NMR (400 MHz, CDCl_3_, *ppm*): δ 8.58 (s, 1H), 8.21-8.19 (m, 1H), 7.97-7.87 (m, 5H), 7.52-7.51 (m, 2H), 7.23 (t, *J* = 8.0 Hz, 2H).

**Synthesis of (4-(9*H*-carbazol-9-yl)phenyl)(dibenzo[b,d]thiophen-2-yl)methanone (SFCOCz)**

Dibenzo[b, d]thiophen-2-yl(4-fluorophenyl)methanone (2.0 g, 6.5 mmol), carbazole (1.6 g, 10.0 mmol) and potassium tert-butoxide (*t*-BuOK) (1.47 g, 13.1 mmol) were dissolved in anhydrous N,N-dimethylformamide (DMF) (50 mL) under argon protection. The reaction solution was allowed to be stirred at room temperature for 15 min, then allowed heated to 110^o^C and stirred overnight. The reaction was quenched with water (25 mL) and extracted with CH_2_Cl_2_ for three times (3×200 mL). The organic layer was collected and dried with anhydrous sodium sulfate (Na_2_SO_4_). After the evaporation of the solvent, the solid residue was purified by column chromatography. Yield: 2.95 g of yellow powder (68%). ^1^H NMR (400 MHz, CDCl_3_, *ppm*): δ 8.73 (s, 1H), 8.28 (t, J = 4.0 Hz, 1H), 8.19-8.13 (m, 4H), 8.01 (s, 2H), 7.93 (t, *J* = 4.0 Hz, 1H), 7.79 (d, *J* = 8.0 Hz, 2H), 7.59-7.53 (m, 4H), 7.49 (t, *J* = 4.0 Hz, 2H), 7.37 (t, *J* = 8.0 Hz, 2H). ^13^C NMR (100 MHz, CDCl_3_): δ 195.40, 144.34, 141.68, 140.29, 139.79, 136.38, 135.65, 135.16, 133.81, 131.92, 128.14, 127.57, 126.38, 126.26, 124.99, 123.88, 123.59, 123.03, 122.73, 122.09, 120.65, 120.52, 109.84. HRMS (MALDI-TOF): *m/z* [M^+^] calcd for C_31_H_19_NOS, 453.22; found, 453.56. Elemental analysis (%) for C_31_H_19_NOS: C, 81.96; H, 4.21; N, 3.04; S, 7.04; found: C, 82.09; H, 4.22; N, 3.09; O, 3.53; S, 7.07.


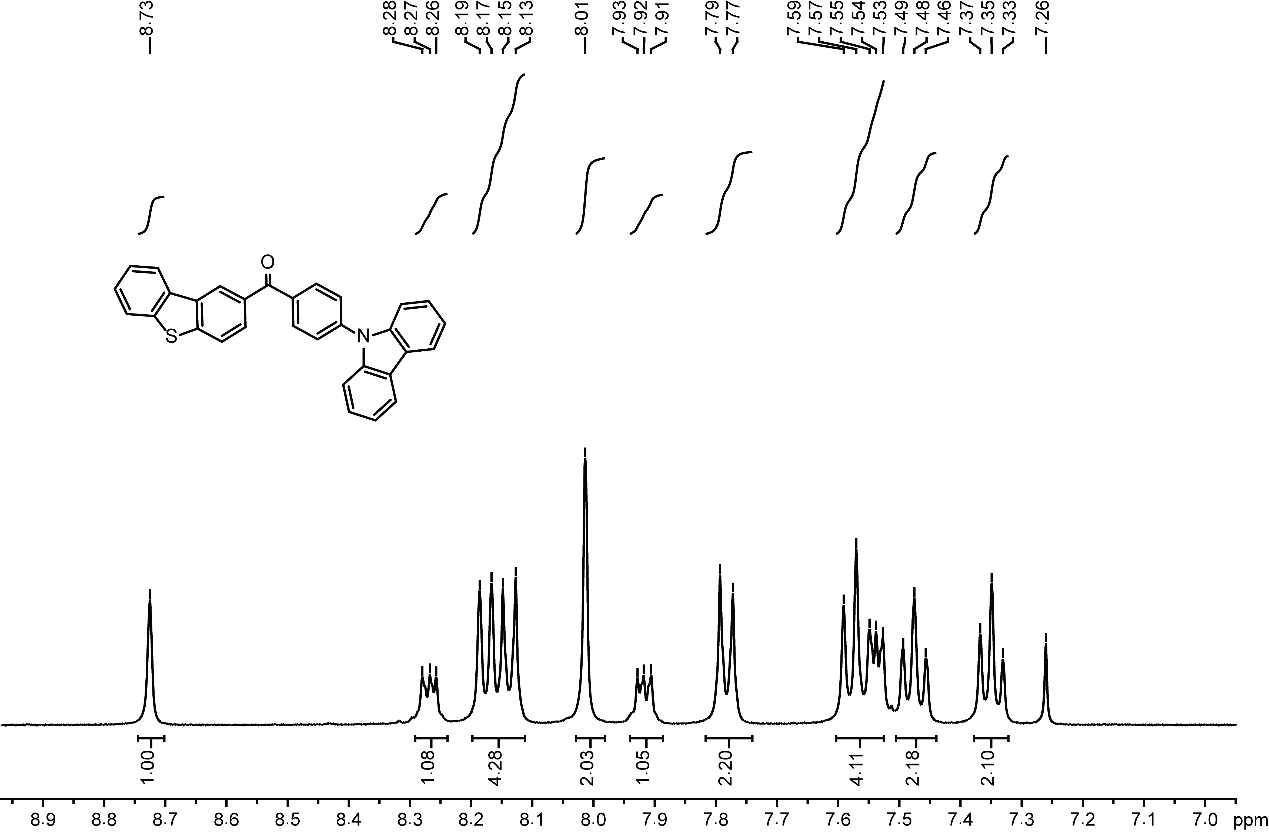


**FIGURE S1.** ^1^H NMR spectrum of **SFCOCz**.


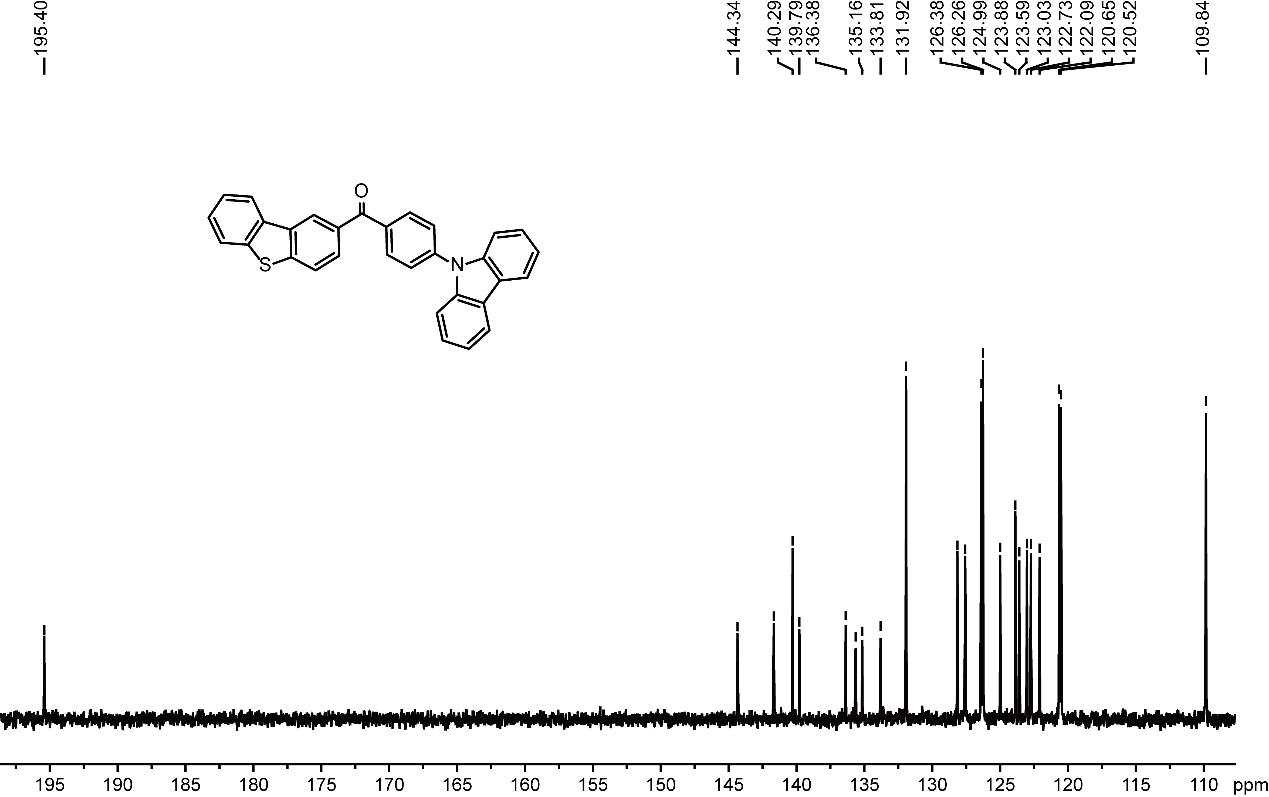


**FIGURE S2.** ^13^C NMR spectrum of **SFCOCz**.

**Synthesis of dibenzo[b,d]thiophen-2-yl(4-(9,9-diphenylacridin-10(9*H*)-yl)phenyl)methanone (SFCODPAC)**

**SFCODPAC** was prepared under the identical synthetic conditions described in the preparation of **SFCOCz** using dibenzo[b,d]thiophen-2-yl (4-fluorophenyl) methanone (2.0 g, 6.5 mmol), 9,9-diphenyl-9,10-dihydroacridine (3.3 g, 10.0 mmol) and potassium tert-butoxide (*t*-BuOK) (1.47 g, 13.1 mmol). Yield: 2.51 g of white powder (62%). ^1^H NMR (400 MHz, CDCl_3_, *ppm*): δ 8.68 (s, 1H), 8.29-8.22 (m, 1H), 8.03-7.89 (m, 5H), 7.54-7.52 (m, 2H), 7.26-7.25 (m, 2H), 7.23-7.22 (m, 6H), 7.15-7.10 (m, 2H), 7.05-6.99 (m, 4H), 6.94 (d, *J* = 4.0 Hz, 4H), 6.55 (d, *J* = 8.0 Hz, 2H). ^13^C NMR (100 MHz, CDCl_3_): δ 195.55, 146.21, 144.87, 144.39, 141.80, 139.76, 137.38, 135.63, 135.12, 133.64, 132.33, 131.05, 130.40, 130.34, 130.16, 128.17, 127.69, 127.56, 126.96, 126.39, 124.98, 123.54, 123.00, 122.67, 122.08, 120.70, 114.36, 56.85. HRMS (MALDI-TOF): m/z [M+] calcd for C_44_H_29_NOS, 620.62; found, 619.78. Elemental analysis (%) for C_44_H_29_NOS: C, 85.21; H, 4.76; N, 2.22; S, 5.12; found: C, 85.27; H, 4.72; N, 2.26; O, 2.58; S, 5.17.


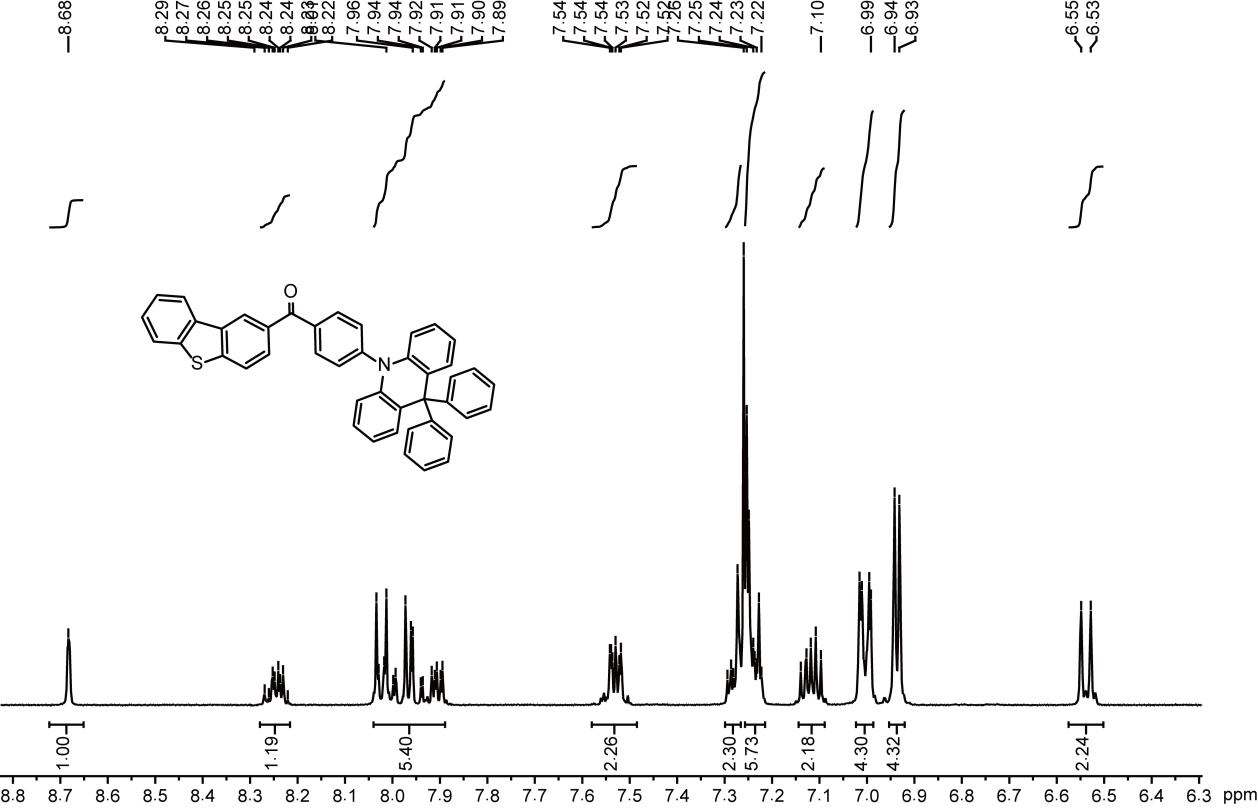


**FIGURE S3.** ^1^H NMR spectrum of **SFCODPAC**.


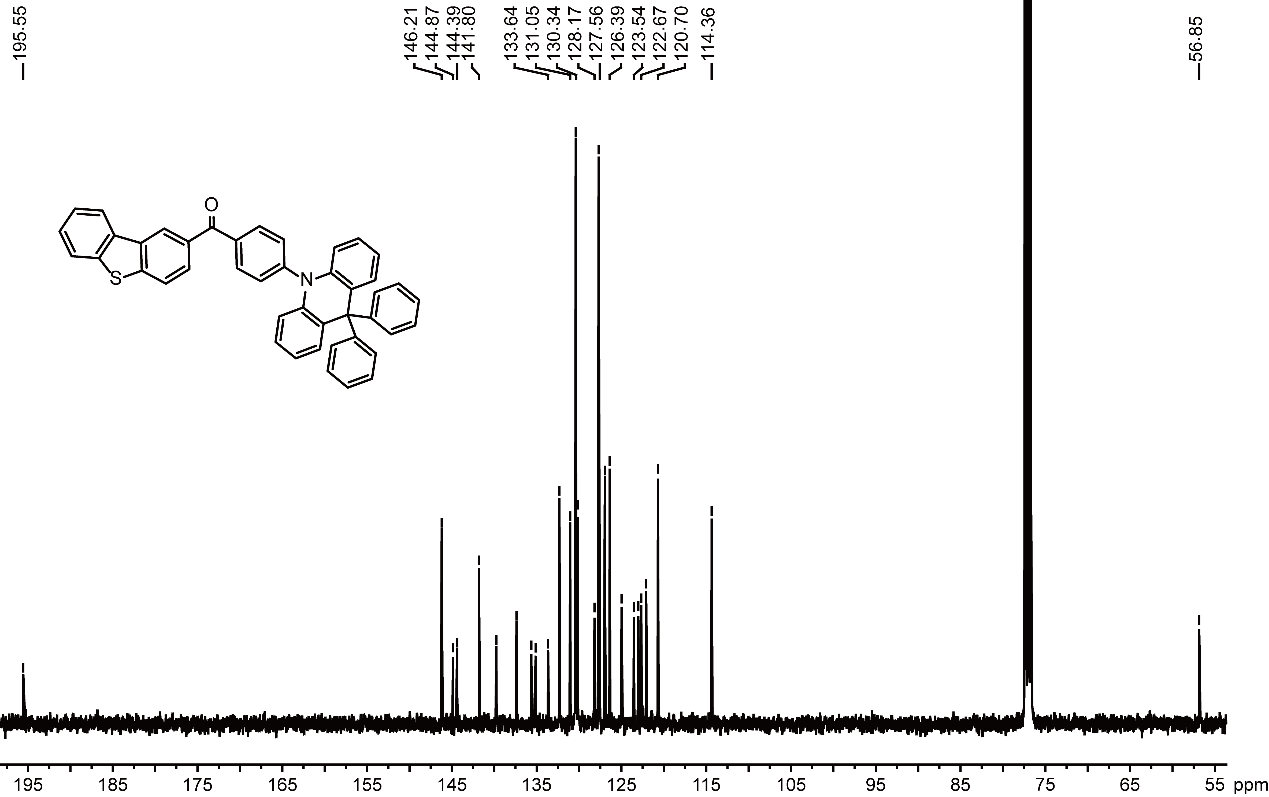


**FIGURE S4.** ^13^C NMR spectrum of **SFCODPAC**.

# Single Crystal Analysis

Single crystals were grown by slow evaporation of a combined CH_2_Cl_2_ and ethanol solution at room temperature. X-ray crystallography was carried out on a Bruker SMART APEX-II CCD diffractometer with graphite monochromated Mo-Kα radiation at 296 K. The crystal structures were analyzed by Diamond 3.2 software and the structure data were summarized in **Table S1**.

**TABLE S1.** Crystallographic data of **SFCOCz** and **SFCODPAC** single crystals.

| Compound | SFCOCz | SFCODPAC |
| --- | --- | --- |
| Empirical formula | C_31_H_19_NOS | C_44_H_29_NOS |
| Formula weight (g mol^-1^) | 453.53 | 619.74 |
| Crystal color | colorless | colorless |
| Wavelength (Å) | 0.71073 | 0.71073 |
| Crystal system | Monoclinic | Monoclinic |
| Space group | P 1 21/n 1 | P 1 21/n 1 |
| *a*, (Å) | 9.696 (4) | 17.127 (3) |
| *b*, (Å) | 8.475 (4) | 9.8337 (18) |
| *c*, (Å) | 27.467 (12) | 19.670 (4) |
| *α*, (deg) | 90 | 90 |
| *β*, (deg) | 92.895 (12) | 105.023 (4) |
| *γ*, (deg) | 90 | 90 |
| volume, (Å^3^) | 2254.3 (17) | 3199.6 (10) |
| *Z* | 4 | 4 |
| Density, (g cm^-3^) | 1.336 | 1.287 |
| *μ*, (mm^-1^) | 0.169 | 0.139 |
| *F* (000) | 944 | 1296 |
| *h*_max_, *k*_max_, *l*_max_ | 13,11,38 | 22,13,17 |
| CCDCNO. | 1964730 | 1964747 |

# Thermal Properties

Thermogravimetric analysis (TGA) and differential scanning calorimetry (DSC) were performed to investigate the thermal properties of the compounds. TGA measurements were conducted on a Netzsch STA2500 thermogravimetric analyses at a heating rate of 10ºC min^-1^ and a nitrogen flow rate of 50 cm^3^ min^-1^. DSC analyses were performed on a Netzsch DSC214 Polyma instrument under a heating rate of 10ºC min^-1^ and a nitrogen flow rate of 20 cm^3^ min^-1^.


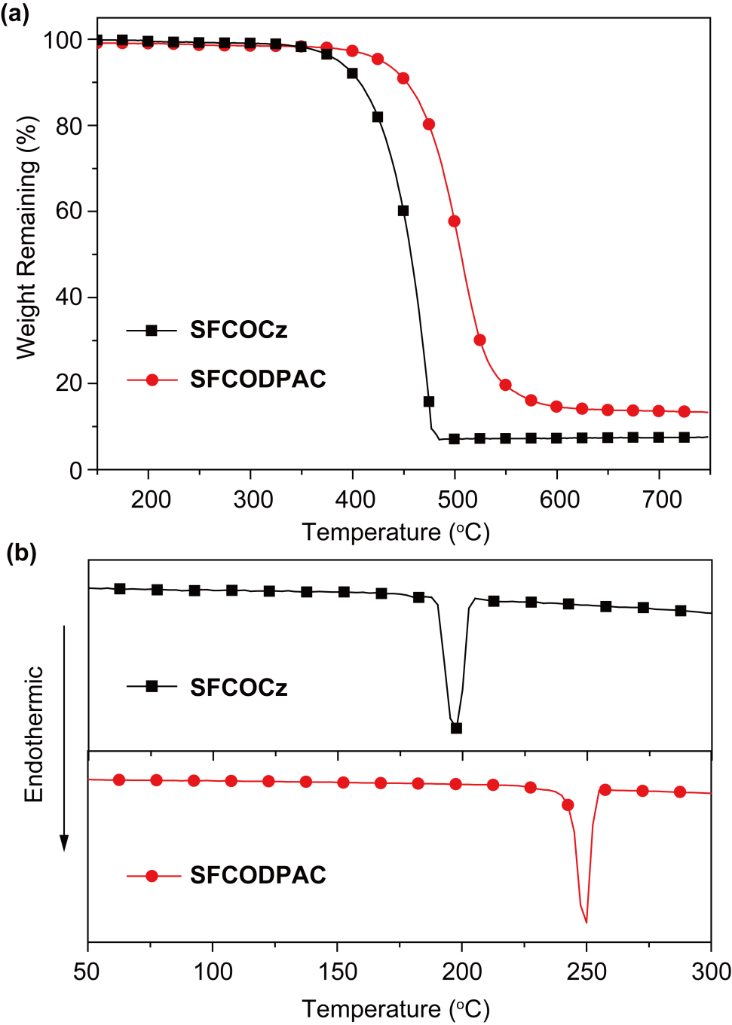


**FIGURE S5.** (a) TGA and (b) DSC curves of **SFCOCz** and **SFCODPAC**.

# Morphology Properties

Atomic force microscopy (AFM) measurements were carried out at room temperature using a Bruker Dimension Icon AFM equipped with Scanasyst-Air peak force tapping mode AFM tips from Bruker. The thin films were prepared through vacuum-deposition on glass substrates under the identical conditions as that in thermally activated delayed fluorescence organic light emitting diode (TADF OLED) device fabrication.


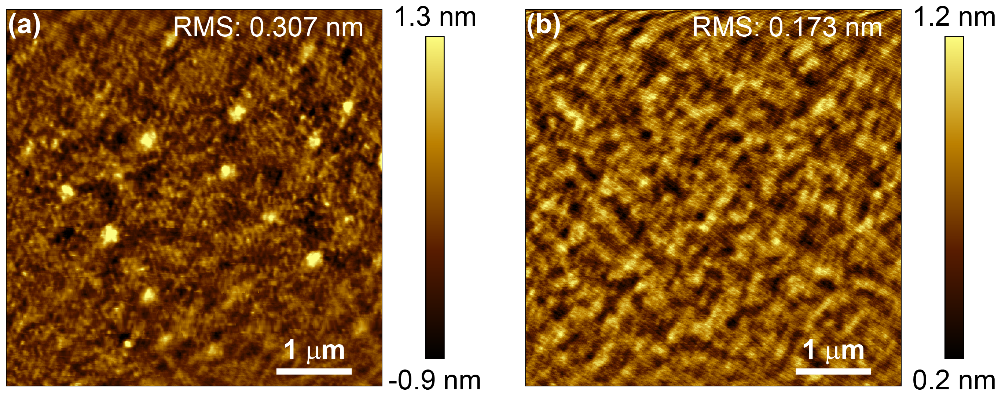


**FIGURE S6**. AFM height images and root-mean-square roughness (RMS) of vacuum-evaporated thin films of (a) **SFCOCz** and (b) **SFCODPAC** on glass substrates

.

# Photophysical Properties

Ultraviolet-visible (UV-Vis) and fluorescence spectra were recorded on a Jasco V-750 spectrophotometer and Edinburgh FLS980, respectively. The phosphorescence spectra of the compounds in CH_2_Cl_2_ were measured using a time-resolved Edinburgh FLS980 fluorescence spectrophotometer at 77 K, with a 5 ms delay time after the excitation (*λ*=290 nm) using a microsecond flash lamp. The concentrations of the compound solutions (in CH_2_Cl_2_) were adjusted to be about 1×10^-5^ mol L^-1^. The thin solid films made for optical property measurements were prepared by casting solution of the compounds on quartz substrates. The lifetimes (*τ*) of the luminescence were obtained by fitting the luminescent intensity decay curve (*I*(t)) with a multi-exponential decay function of

................................(S1)

where *A_i_* and *τ_i_* represent the amplitudes and lifetimes of the individual components for multi-exponential decay profiles, respectively.


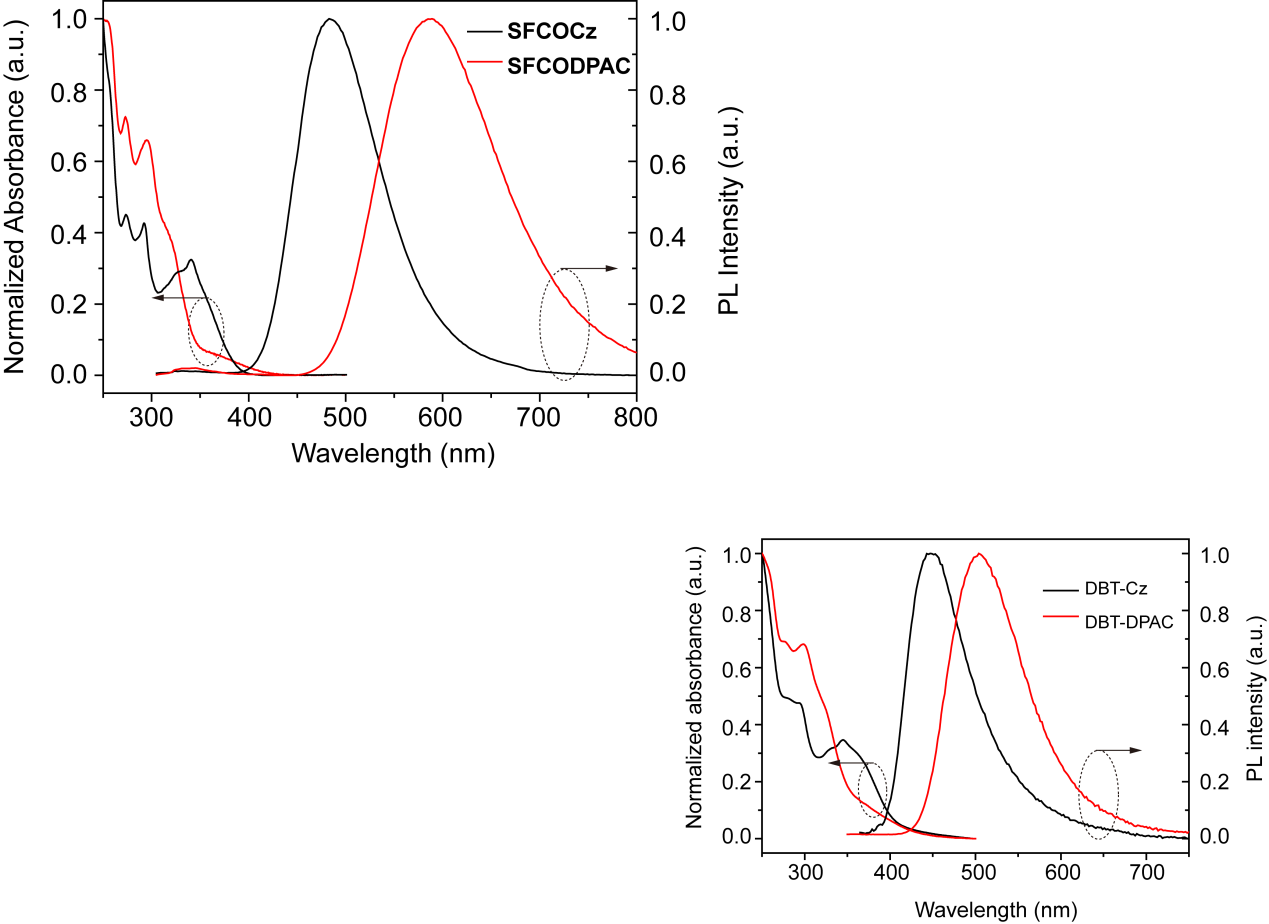


**FIGURE S7.** UV-vis absorption and photoluminescence (PL) spectra of **SFCOCz** and **SFCODPAC** in dichloromethane (CH_2_Cl_2_) solution (~10^-5^ M).


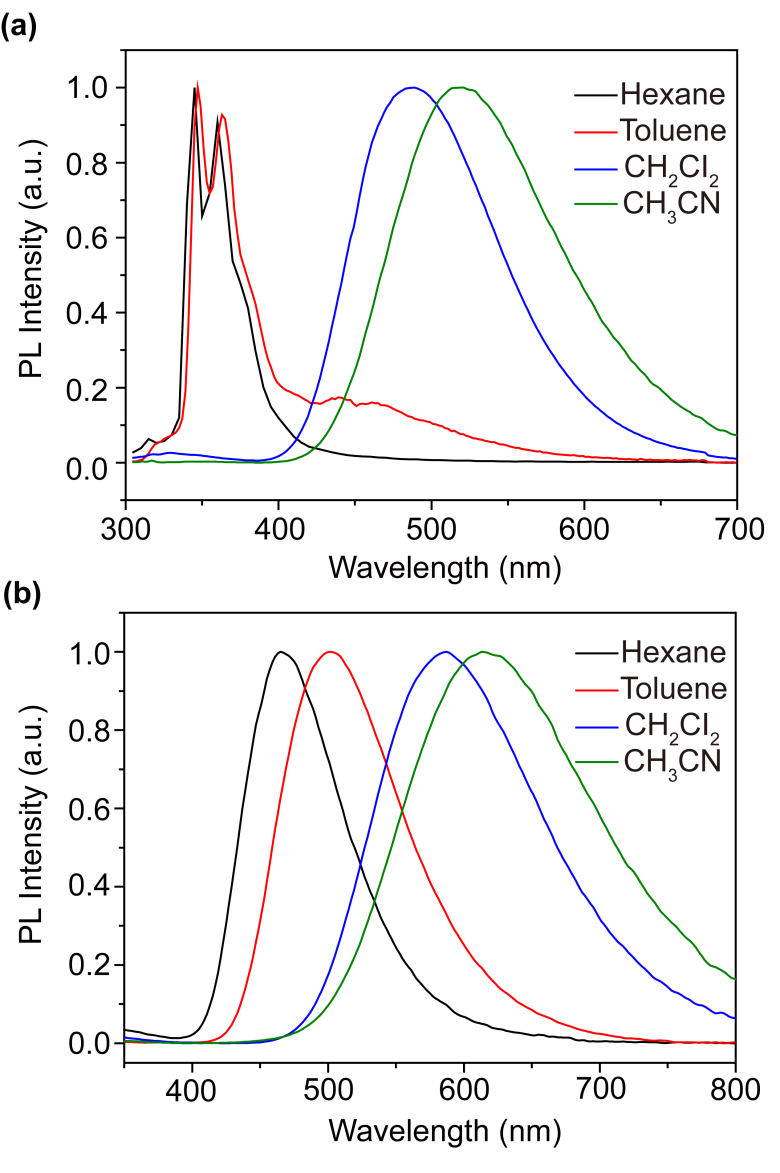


**FIGURE S8.** PL spectra of (a) **SFCOCz** and (b) **SFCODPAC** in different solvents (~10^-5^ M).


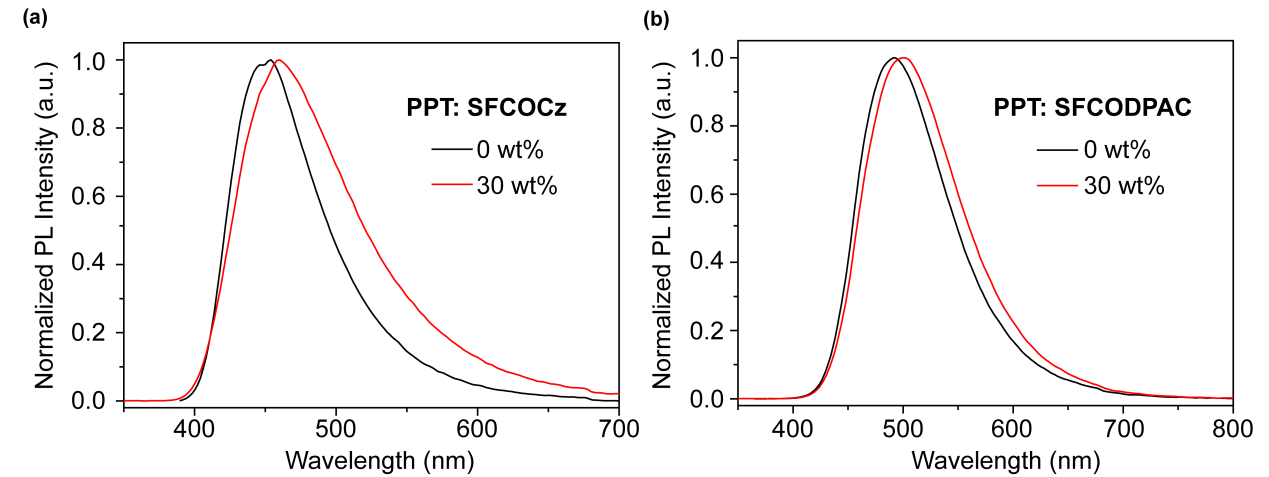


**FIGURE S9**. PL spectra of (a) **SFCOCz** and (b) **SFCODPAC** doped in PPT film with different doping concentrations (wt%).


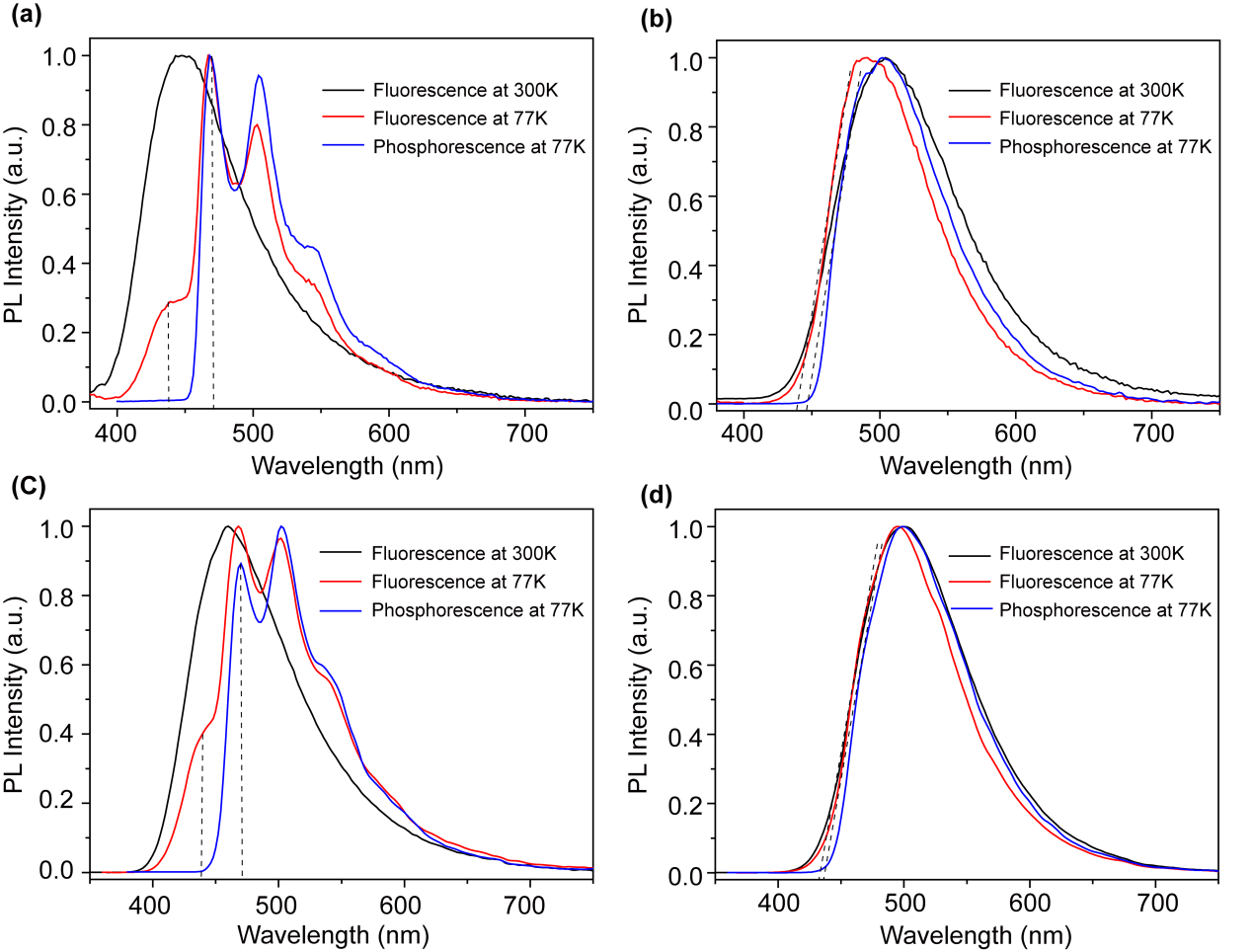


**FIGURE S10.** Fluorescence and phosphorescence spectra of neat (a, b) and non-doped (c, d) film of (a, c) **SFCOCz**, (b, d) **SFCODPAC** neat film, (c) PPT: 30wt% **SFCOCz**, (d) PPT: 30wt% **SFCODPAC**.

**
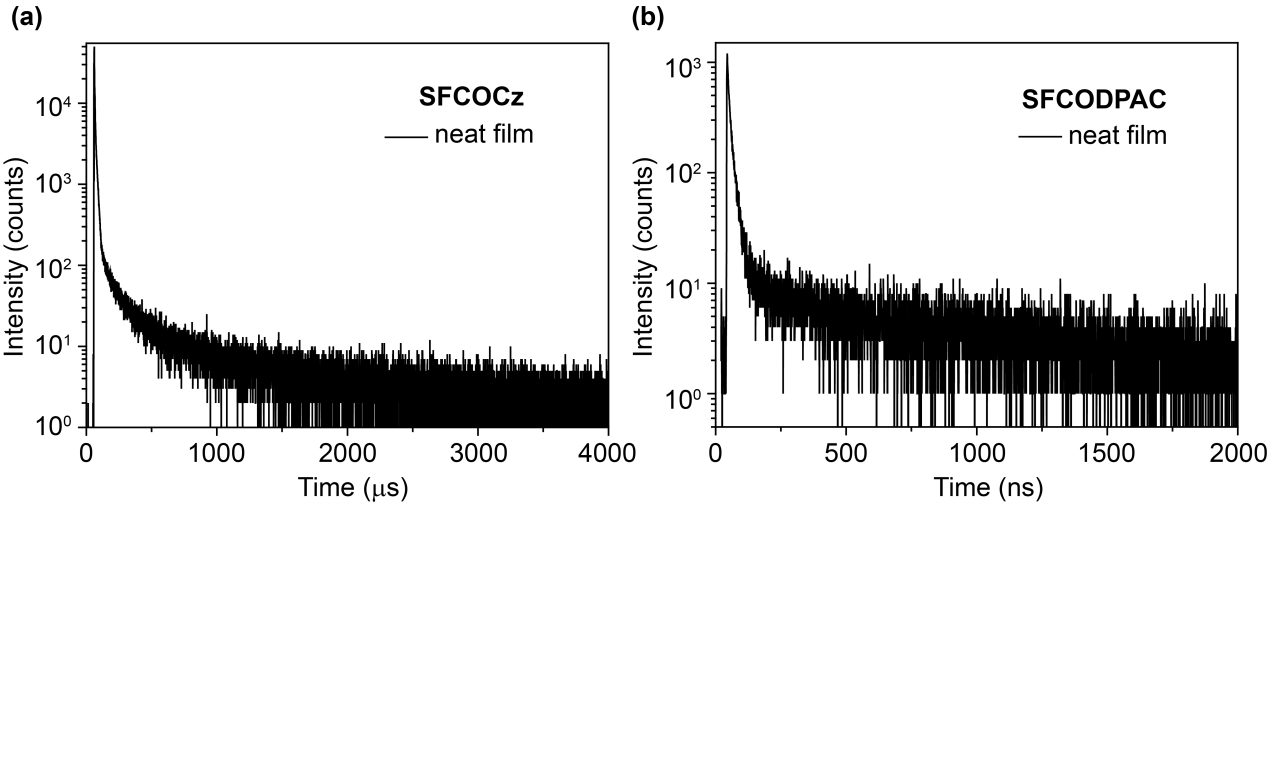
**

**FIGURE S11**. Transient PL decay curve of the neat film of **SFCOCz** (a) and **SFCODPAC** (b).

# Calculation of the Photophysical Rate Constants.

The quantum efficiencies and rate constants were determined using the following equations (**S2-S12** (Zhang et al., 2014))

$\Phi_{prompt}=\Phi_{PL}R_{prompt}$ (S2)

$\Phi_{delayed}=\Phi_{PL}R_{delayed}$ (S3)

$k_{F}=\Phi_{prompt}/\tau_{prompt}$ (S4)

$\Phi_{PL}=k_{F}/\left(k_{F}+k_{IC}\right)$ (S5)

$\Phi_{prompt}=k_{F}/\left(k_{F}+k_{IC}+k_{ISC}\right)$ (S6)

$\Phi_{IC}=k_{IC}/\left(k_{F}+k_{IC}+k_{ISC}\right)$ (S7)

$\Phi_{ISC}=k_{ISC}/\left(k_{F}+k_{IC}+k_{ISC}\right)=1-\Phi_{prompt}-\Phi_{IC}$ (S8)

$\Phi_{\text{RISC}}=\Phi_{delayed}/\Phi_{ISC}$ (S9)

$k_{RISC}=\left(k_{p}k_{d}\Phi_{delayed}\right)/\left(k_{ISC}\Phi_{prompt}\right)$ (S10)

$k_{p}=1/\tau_{prompt}$ (S11)

$k_{\text{d}}\text{=1/}\tau_{\text{delayed}}$ (S12)

**TABLE S2.** Photophysical profiles of neat films and doped films in PPT (30 wt%) of **SFCOCz** and **SFCODPAC**^a)^.

|  | **SFCOCz** | |  | **SFCODPAC** | |
| --- | --- | --- | --- | --- | --- |
|  | neat film | 30 wt% |  | neat film | 30 wt% |
| *λ*_em_ [nm] | 450 | 458 |  | 492 | 500 |
| *Φ*_PL_ [%] | 22.14 | 8.09 |  | 73.10 | 53.96 |
| *Φ*_prompt_ [%] | 12.02 | 3.10 |  | 56.30 | 29.74 |
| *Φ*_delayed_ [%] | 10.12 | 4.99 |  | 17.53 | 24.22 |
| *Φ*_ISC_ [%] | 45.72 | 61.65 |  | 22.98 | 44.89 |
| *Φ*_RISC_ [%] | 22.14 | 8.09 |  | 76.28 | 53.95 |
| τ_prompt_ [ns] | 5.71 | 4.84 |  | 14.20 | 15.48 |
| τ_delayed_ [us] | 332.91 | 20.12 |  | 0.22 | 0.84 |
| *k*_F_ [×10^6^ s^-1^] | 21.05 | 6.41 |  | 39.65 | 19.21 |
| *k*_IC_ [×10^6^ s^-1^] | 74.01 | 72.83 |  | 14.60 | 16.39 |
| *k*_ISC_ [×10^6^ s^-1^] | 80.07 | 127.38 |  | 16.18 | 29.00 |
| *k*_RISC_ [×10^6^ s^-1^] | 0.01 | 0.13 |  | 6.16 | 2.16 |
| Δ*E*_ST_ [eV] | 0.17 | 0.21 |  | 0.03 | 0.02 |

^a)^ Abbreviations: *Φ*_PL_ = absolute photoluminescence quantum yield; τ_prompt_ and τ_delayed_ = lifetimes calculated from transient fluorescence decay profiles; *Φ*_prompt_ and *Φ*_delayed_ = prompt and delayed components, respectively; *Φ*_ISC_ = the intersystem crossing quantum yield; *k*_F_ = fluorescence decay rate; *k*_IC_ = internal conversion decay rate; *k*_ISC_ = intersystem crossing rate; *k*_RISC_ = reverse intersystem crossing rate; Δ*E*_ST_ = singlet and triplet splitting energy.

# Theoretical Calculations

Theoretical calculations were performed on Gaussian 09 program with the Becke’s three-parameter exchange functional along with the Lee-Yang Parr’s correlation functional (B3LYP) using def2sv basis sets (Yin et al., 2010). The geometries at the ground state (S_0_) and the lowest triplet excited state (T_1_) were fully optimized by spin-restricted and spin-unrestricted DFT calculations at the B3LYP/def2sv level, respectively. The optimized geometry at the lowest singlet excited state (S_1_) was obtained by TD-DFT calculations at the same functional and basis set. To get further insights into the nature of the excited states, natural transition orbitals (NTOs) analysis was performed based on TD-DFT results to offer a compact orbital representation for the electronic transition density matrix. Vibrational frequency calculations were subsequently carried out to confirm that all these optimized structures are corresponding to the minima on the potential energy surfaces. Using the overlap integral function embedded in Multiwfn, the overlap between HOMO (*φ*_H_) and LUMO (*φ*_L_) of a molecule can be calculated according to **Equation** **S13.** Similarly, the overlap extent between the highest occupied natural transition orbitals (HONTOs) and the lowest unoccupied natural transition orbitals (LUNTOs) at both S_1_ (*I*_S_) and T_1_ (*I*_T_) states:

*I*H/L = ∫ *ϕH* (*r*) *ϕL* (*r*) *dr* (S13)

# Electrochemical Properties

Cyclic voltammetry (CV) measurements were performed at room temperature on a CHI660E system in a typical three-electrode cell with a working electrode (glass carbon), a reference electrode (Ag/Ag^+^), referenced against ferrocene/ferrocenium (FOC) and a counter electrode (Pt wire). The electrochemical experiments were carried out in an acetonitrile solution of Bu_4_NPF_6_ (0.1 M) at a sweeping rate of 100 mV s^-1^. The highest occupied molecular orbital (HOMO) energy levels (*E*_HOMO_) of the materials deposited as thin films on the surface of the working electrode were measured according to the reference energy level of ferrocene (4.8 eV below the vacuum) as illustrated in **Equation S14**:

*E*_HOMO_ = -[ - *E*_(_*_F_*_c/_*_F_*_c+)_ + 4.8 ] eV (S14)

where *E*_(Fc/Fc+)_ is the onset oxidative voltage of FOC vs Ag/Ag^+^ and is the onset potential of the oxidation wave. The lowest unoccupied molecular orbital (LUMO) energy level (*E*_LUMO_) was estimated by adding the optical band-gap (*E*_g_) to the corresponding HOMO energy level as in **Equation S15**:

*E*_LUMO_ = [*E*_HOMO_ + *E*_g_] eV (S15)


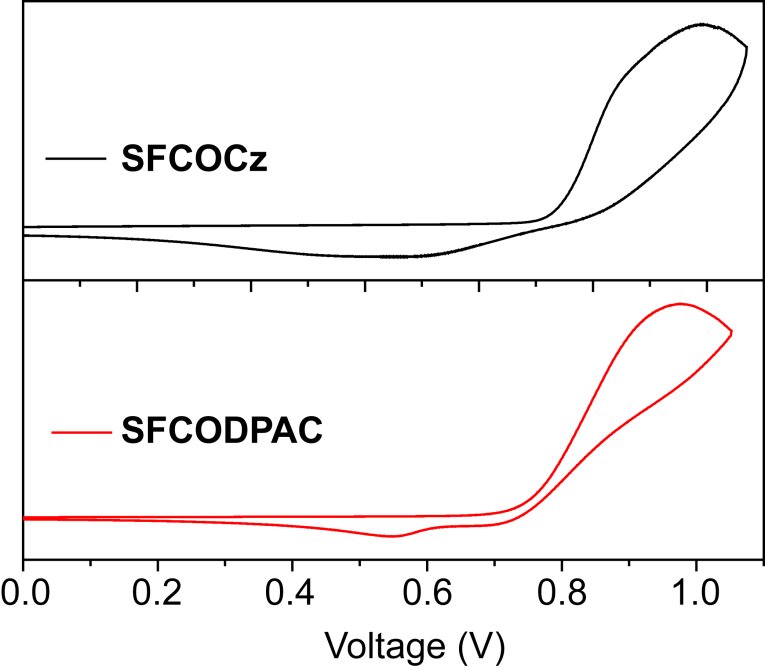


**FIGURE S12.** Cyclic voltammograms of **SFCOCz** and **SFCODPAC** films coated on the working electrodes.

# Device Fabrications and Measurements

Typically, ITO-coated glass substrates were etched, patterned, and washed by ultrasonic with detergent, deionized water, acetone, and ethanol in turn. Organic layers were deposited by high-vacuum (10^-6^ Torr) thermal evaporation at a rate of 0.1-0.2 nm s^-1^. The layer thickness and the deposition rate were monitored in situ by an oscillating quartz thickness monitor. The devices were measured after fabrication without encapsulation under ambient atmosphere at room temperature. The layer thickness and deposition rate (1~2 Å s^-1^) were monitored *in situ* by an oscillating quartz thickness monitor. Electroluminescent (EL) spectra of the devices were measured by a PR655 spectra scan spectrometer. The luminance-voltage and current-voltage characteristics were measured simultaneously with an optical power meter and a Keithley 2400 voltage current source. And the external quantum efficiency (EQE) (Tao et al., 2016) was calculated by the following **Equation** **S16**:

$EQE=\frac{\pi e\eta_{cd/A}\int\lambda p(\lambda)d\lambda}{hcK_{m}\int p(\lambda)\Phi(\lambda)d\lambda}$ (S16)

where *η*_cd/A_ is the current efficiency (cd/A); *h* is the Planck constant; *c* is the speed of light in vacuum; λ is the wavelength (nm); *e* is the electron charge; *p(λ*) is relative electroluminescent intensity at each wavelength; Φ(*λ*) is the Commision International de l’Eclairage chromaticity (CIE) standard photopic luminous efficiency function; and *K_m_* is a constant of 683 lm/W.

**TABLE S3.** Device performance of **SFCOCz** and **SFCODPAC**.

| Device | *λ*_EL_ (nm)^a^ | V_on_ (V)^b^ | Maximum values | | CIE^e^ | Values at 100 cd m^-2^ |  | Values at 1000 cd m^-2^ |
| --- | --- | --- | --- | --- | --- | --- | --- | --- |
|  |  |  | Efficiency^c^ | L_max_ (cd m^-2^)^d^ |  | Efficiency^c^ |  | Efficiency^c^ |
| TA | 451 | 4.8 | 0.3, 0.2, 0.3 | 529 | (0.16, 0.11) | 0.16, 0.05, 0.19 |  | - |
| TB | 482 | 4.4 | 16.8, 9.1, 7.5 | 5598 | (0.20, 0.37) | 16.7, 7.5, 7.4 |  | 12.7, 3.7, 5.6 |
| TC | 468 | 4.8 | 0.8, 0.5, 0.6 | 672 | (0.15, 0.18) | 0.28, 0.09, 0.22 |  | - |
| TD | 485 | 3.8 | 35.6, 29.8, 15.9 | 11595 | (0.19, 0.37) | 29.1, 11.8, 13.1 |  | 21.0, 5.6, 9.4 |

^a^ EL peak values measured at 10 V; ^b^ In the order of onset; ^c^ In the order of CE (cd A^-1^), PE (lm W^-1^) and EQE (%); ^d^ L=luminance; ^e^ Commission Internationale de L'Eclairage.

**TABLE S4. Electroluminescent performance of OLEDs based on AIE type TADF emitters.**

| Emitter | λ_EL_  [nm] | L_max_  [cd m^−2^] | CE_max_/PE_,max_/EQE_,max_  [cd A^−1^/lm W^−1^/%] | CIE | Ref. |
| --- | --- | --- | --- | --- | --- |
| CDE1 | 552 | >10000 | ≈45/≈20/13.8 | (0.40, 0.54) | (Li et al., 2016) |
| DBT-BZ-MAC | 516 | 27270 | 43.3/35.7/14.2 | (0.26, 0.55) | (Guo et al., 2017) |
| CP-BP-DMAC | 502 | 37680 | 41.6/37.9/15.0 | (0.23, 0.49) | (Huang et al., 2017) |
| DBQ-3DMAC | 548 | 29843 | 41.2/45.4/12.0 | (0.40, 0.57) | (Yu et al., 2018) |
| *o*-ACSO2 | 492 | ≈1000 | 14.1/7.8/5.9 | (0.23, 0.40) | (Wu et al., 2018) |
| CCDD | 543 | 14600 | 39.8/41.7/12.7 | (0.39, 0.56) | (Zhao et al., 2017) |
| PTSOPO | ≈530 | >10000 | –/–/17.0 | - | (Lee et al., 2016) |
| DCPDAPM | 522 | 123371 | 26.9/15.6/8.2 | (0.28, 0.59) | (Zhao et al., 2018) |
| tBuG2B | ≈500 | 3029 | 22.5/17.2/8.9 | (0.25, 0.48) | (Matsuoka et al., 2018) |
| BP-TXDMAc | 494 | 6823 | 34.7/30.1/14.2 | (0.20, 0.39) | (Wang et al., 2019) |
| PTZ-AD | 549 | 47450 | 50.48/60.04/17.08 | (0.42, 0.55) | (Xiang et al., 2019) |
| TAT-2BP | 530 | - | 32.3/33.0/9.8 | - | (Liu et al., 2019) |

# References

Guo, J., Li, X., Nie, H., Luo, W., Gan, S., Hu, S.et al. (2017). Achieving high-performance nondoped OLEDs with extremely small efficiency roll-off by combining aggregation-induced emission and thermally activated delayed fluorescence. *Adv. Funct. Mater.* 27, 1606458. doi: 10.1002/adfm.201606458Huang, J., Nie, H., Zeng, J., Zhuang, Z., Gan, S., Cai, Y.et al. (2017). Highly efficient nondoped OLEDs with negligible efficiency roll-off fabricated from aggregation-induced delayed fluorescence luminogens. *Angew. Chem. Int. Ed.* 56, 12971-12976. doi: 10.1002/anie.201706752

Lee, I.H., Song, W., Lee, J.Y. (2016). Aggregation-induced emission type thermally activated delayed fluorescent materials for high efficiency in non-doped organic light-emitting diodes. *Org. Electron.* 29, 22-26. doi: 10.1016/j.orgel.2015.11.019

Li, Y., Xie, G., Gong, S., Wu, K., Yang, C. (2016). Dendronized delayed fluorescence emitters for non-doped, solution-processed organic light-emitting diodes with high efficiency and low efficiency roll-off simultaneously: two parallel emissive channels. *Chem. Sci.* 7, 5441-5447. doi: 10.1039/C6SC00943C

Liu, Y., Wu, X., Chen, Y., Chen, L., Li, H., Wang, W.et al. (2019). Triazatruxene-based thermally activated delayed fluorescence small molecules with aggregation-induced emission properties for solution-processable nondoped OLEDs with low efficiency roll-off. *J. Mater. Chem. C.* 7, 9719-9725. doi: 10.1039/c9tc02927c

Matsuoka, K., Albrecht, K., Nakayama, A., Yamamoto, K., Fujita, K. (2018). Highly efficient thermally activated delayed fluorescence organic light-emitting diodes with fully solution-processed organic multilayered architecture: impact of terminal substitution on carbazole–benzophenone dendrimer and interfacial engineering. *ACS Appl. Mater. Interface* 10, 33343-33352. doi: 10.1021/acsami.8b09451

Tao, Y., Xu, L., Zhang, Z., Chen, R., Li, H., Xu, H., et al. (2016). Achieving optimal self-adaptivity for dynamic tuning of organic semiconductors through resonance engineering. *J. Am. Chem. Soc.* 138, 9655-9662. doi: 10.1021/jacs.6b05042

Wang, L., Cai, X., Li, B., Li, M., Wang, Z., Gan, L.et al. (2019). Achieving enhanced thermally activated delayed fluorescence rates and shortened exciton lifetimes by constructing intramolecular hydrogen bonding channels. *ACS Appl. Mater. Interface* 11, 45999-46007. doi: 10.1021/acsami.9b16073

Wu, K., Wang, Z., Zhan, L., Zhong, C., Gong, S., Xie, G.et al. (2018). Realizing highly efficient solution-processed homojunction-like sky-blue OLEDs by using thermally activated delayed fluorescent emitters featuring an aggregation-induced emission property. *J. Phys. Chem. Lett.* 9, 1547-1553. doi: 10.1021/acs.jpclett.8b00344

Xiang, S., Guo, R., Huang, Z., Lv, X., Sun, S., Chen, H.et al. (2019). Highly efficient yellow nondoped thermally activated delayed fluorescence OLEDs by utilizing energy transfer between dual conformations based on phenothiazine derivatives. *Dyes Pigments* 170, 107636. doi: 10.1016/j.dyepig.2019.107636

Yin, J., Zhang, S., Chen, R., Ling, Q., Huang, W. (2010). Carbazole endcapped heterofluorenes as host materials: theoretical study of their structural, electronic, and optical properties. *Phys. Chem. Chem. Phys.* 12, 15448. doi: 10.1039/c0cp00132e

Yu, L., Wu, Z., Xie, G., Zeng, W., Ma, D., Yang, C. (2018). Molecular design to regulate the photophysical properties of multifunctional TADF emitters towards high-performance TADF-based OLEDs with EQEs up to 22.4% and small efficiency roll-offs. *Chem. Sci.* 9, 1385-1391. doi: 10.1039/C7SC04669C

Zhang, Q., Kuwabara, H., Potscavage, W.J., Huang, S., Hatae, Y., Shibata, T., et al. (2014). Anthraquinone-based intramolecular charge-transfer compounds: computational molecular design, thermally activated delayed fluorescence, and highly efficient red electroluminescence. *J. Am. Chem. Soc.* 136, 18070-18081. doi: 10.1021/ja510144h

Zhao, H., Wang, Z., Cai, X., Liu, K., He, Z., Liu, X.et al. (2017). Highly efficient thermally activated delayed fluorescence materials with reduced efficiency roll-off and low on-set voltages. *Mater. Chem. Front.* 1, 2039-2046. doi: 10.1039/C7QM00195A

Zhao, Y., Wang, W., Gui, C., Fang, L., Zhang, X., Wang, S.et al. (2018). Thermally activated delayed fluorescence material with aggregation-induced emission properties for highly efficient organic light-emitting diodes. *J. Mater. Chem. C.* 6, 2873-2881. doi: 10.1039/C7TC04934J
